# Vascular Atypia in a Cardiac Myxoma Patient

**DOI:** 10.7759/cureus.18646

**Published:** 2021-10-10

**Authors:** Meaghan Standridge, Jay Gaucher, Chelsea M Burgin

**Affiliations:** 1 Department of Surgery, University of Tennessee Medical Center, Knoxville, USA; 2 Department of Medicine, Prisma Health, Greenville, USA; 3 Department of Emergency Medicine, Prisma Health, University of South Carolina School of Medicine, Greenville, USA

**Keywords:** transesophageal echo, cardiac echo, angioma, myocardial revascularization, myxoma, head and neck mri/mra, cardiac catheterization, cardiac tumor, cardiac mass, acute stroke

## Abstract

Cardiac masses can manifest as a variety of signs and symptoms in adults. In this report, we report a case that highlights the value of advanced imaging of a newly symptomatic and previously undiagnosed cardiac neoplasm. In addition to the standard transthoracic and transesophageal echocardiography, cardiac catheterization may be employed to further understand the vascularity of such cardiac pathology prior to surgical intervention.

## Introduction

Cardiac myxomas are the most common among benign cardiac tumors, and they typically present in females between the ages of 30 and 60 years [1-3]. Myxomas are benign gelatinous neoplasms; however, they are commonly accompanied by significant morbidity and mortality secondary to complications such as acute intracranial infarct due to emboli [1]. Dual coronary artery supply can occur, typically from the left circumflex artery and right coronary artery [4], which highlights the necessity of thorough investigation with advanced imaging prior to surgical resection.

## Case presentation

A 65-year-old female presented to the emergency department with a chief complaint of difficulty in walking and talking. Her symptoms had begun two days prior, following a syncopal episode. She recalled waking up on the bathroom floor with new urinary incontinence. She reported generalized weakness and expressive aphasia. Pertinent past medical history was positive for adolescent syncope but negative for seizure.

On physical examination, her vitals were reassuring. She had right-sided facial droop, right-sided weakness in the upper and lower extremity as well as mild dysarthria. Her National Institutes of Health Stroke Scale (NIHSS) score was 4/42. Emergency department lab evaluation included a metabolic panel, complete blood count, HbA1C, serial troponins, and urine analysis. Imaging obtained included CT head and chest X-ray. Labs and imaging were all within normal range except for normocytic anemia, hemoglobin of 10.1 g/dl, and hematocrit of 29.3%. She was admitted for acute stroke with plans to perform advanced imaging including an echocardiogram (echo), magnetic resonance angiogram (MRA) of the head and neck as well as MRI of the head.

A likely diagnosis with this presentation is primary ischemic stroke or secondary ischemic stroke. Other medical conditions to consider include aortic stenosis, atrial fibrillation, carotid artery pathology, coronavirus disease 2019 (COVID-19), endocarditis, intracranial hemorrhage, vascular anomaly, or metabolic derangement.

**Figure 1:**
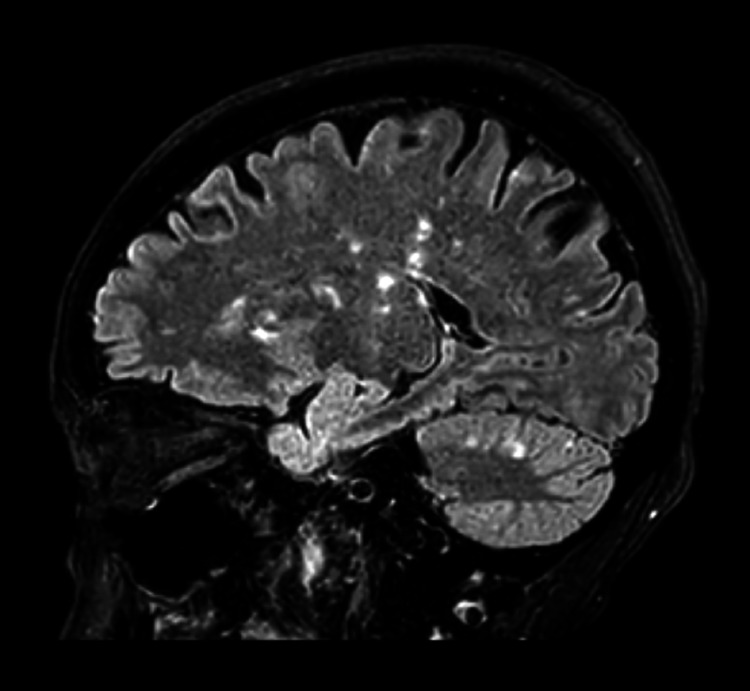
MRI brain with multiple infarctions in both the cerebrum and cerebellum MRI: magnetic resonance imaging

MRA of both the head and neck were negative. However, MRI of the brain without contrast showed numerous acute infarctions in both cerebral and cerebellar hemispheres indicative of emboli or severe hypoperfusion (Figure 1).

**Figure 2:**
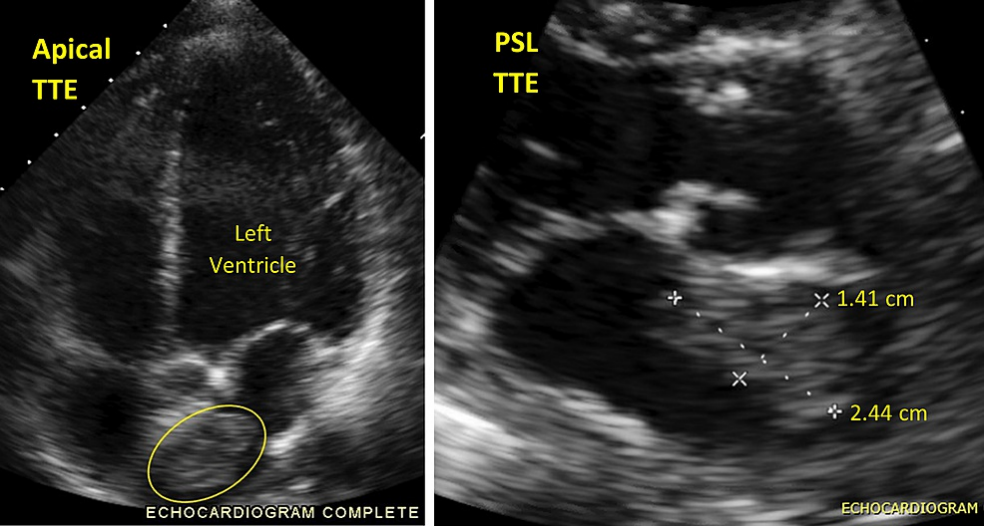
Transthoracic echo Left image: apical four-chamber view with the left atrial mass outlined. Right image: magnified parasternal long axis centering the aortic valve and left atrium with tumor measurements

Transthoracic echo (TTE) was performed, which revealed normal left ventricular diastolic and systolic function as well as right ventricular systolic function. Mild mitral and tricuspid regurgitation was seen in addition to a dense mass in the left atrium measuring 1.41 x 2.44 cm, which is illustrated in Figure 2.

**Figure 3:**
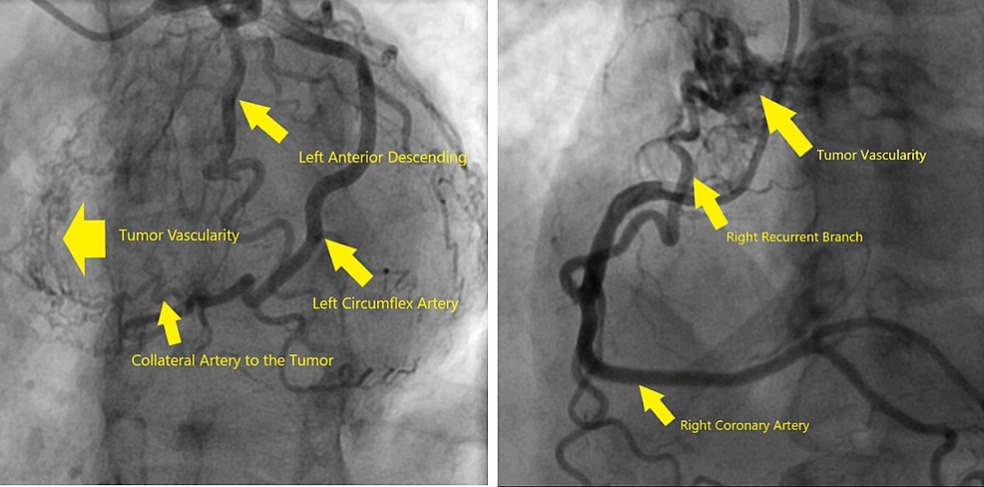
Cardiac catheterization illustrating patent vasculature with collateral vessels and significant neovascularization supplying the tumor

Cardiac catheterization and transesophageal echo (TEE) were added to her workup in preparation for mass resection. On cardiac catheterization, the mass was highlighted by multiple collateral branches from the distal left circumflex artery as well as a large atrial recurrent branch extending from the right coronary artery (Figure 3). Also identified was the active outflow of blood from the tumor itself into the left atrium, seen during angiography and on TEE.

Video 1 shows tumor vascularity on catheterization.

**Video 1:** Tumor vascularity on catheterization

**Figure 4:**
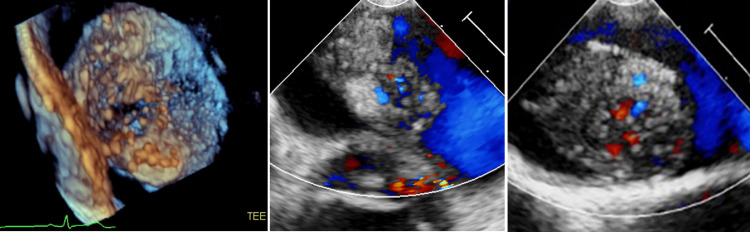
Images from TEE demonstrating irregular borders and varying echogenicity in 3D as well as color flow highlighting vascularity within the tumor TEE: transesophageal echocardiogram

TEE showed a normal-sized left atrium yet demonstrated a large, mobile, multi-lobular mass on the interatrial septum. The mass was irregular with highly mobile components. It also was found to be anechoic in areas where color flow raised the concern for a hemangioma (Figure 4).

 Video 2 shows vascular tumor on TEE.

**Video 2:** Vascular tumor on TEE TEE: transesophageal echocardiogram

The patient underwent surgical excision of the left atrial mass without intraoperative complications. Pathology reported a 3.5 x 3.5 x 1.0-cm atrial myxoma without atypical features. She was discharged to a rehabilitation facility. After one ambulatory post-op appointment with cardiothoracic surgery, she was released and instructed to follow up as indicated with cardiology.

## Discussion

Primary cardiac tumors are rare entities. The prevalence of cardiac tumors is estimated to be 0.02% and the incidence is reported to be 0.1% [1]. Cardiac myxomas are the most common benign tumor, accounting for 50-70% of all cardiac masses [2]. The etiology of a myxoma is currently unknown with only 10% associated with a genetic pattern [1]. They are usually identified in middle-aged women with over 75% of them arising in the left atrium at the border of the fossa ovalis and less than 18% originating in the right atrium [3].

Complications of a myxoma include arrhythmias, emboli, and coronary vascular obstruction. Embolization most commonly involves the cerebral arteries [2,3]. Although the tumor usually has a soft, gelatinous consistency with smooth borders, it can develop superficial thrombus giving rise to embolization. It is estimated that 20-35% of patients with cardiac myxoma will suffer from neurologic complications due to embolization [1]. Myxomas have an average size of 5-6 cm but those up to 15 cm have also been reported [2]. However, size does not appear to be a factor in embolization. Rather, it has to do with the mobility and friability of the tumor that increases the risk of embolization [1]. Arrhythmias are possible, although more likely with fibromas, if the tumor infiltrates a neural pathway. Obstructive symptoms can arise if the tumor is mobile enough to reduce the return of blood to the heart, resulting in paroxysmal heart failure or dyspnea [1].

Neovascularization of atrial myxomas, as seen in this case, is not unusual. The reported prevalence of neovascularization is 37-52% with the most common source being the circumflex artery, followed by the right coronary artery [4]. Unique to this case was the active outflow of blood from the tumor itself into the left atrium, as seen on TEE color flow as well as during angiography. Awareness of the vascular characteristics of a myxoma is essential when preparing for safe resection.

As for distinguishing a cardiac myxoma from hemangioma, hemangiomas consist of dilated vascular channels, on echocardiographic imaging, a mass with echolucencies [5]. Tumor blush is a sign that can be seen in roughly 33% of cardiac hemangiomas on angiography, although it can also be seen in atrial myxomas [6]. Due to the high vascularity of this patient’s tumor and the hypoechoic regions identified on TEE, it was unclear prior to surgical pathology if the mass was a hemangioma or myxoma.

## Conclusions

Although primary cardiac tumors are rare, they often lead to significant morbidity and mortality. Surgical resection is critical in the management of cardiac neoplasms such as myxoma to prevent future complications. Furthermore, appropriate imaging is essential to understand the complexity of the vascular supply of a tumor to aid in judicious surgical intervention. Follow-up pathology can be useful in determining the final diagnosis.
